# Population-Level Dissemination of a Smoking Cessation Smartphone App: Quasi-Experimental Comparison of Values-Based Messages in Social Media Advertisements

**DOI:** 10.2196/71619

**Published:** 2025-07-28

**Authors:** Jonathan B Bricker, Margarita Santiago-Torres, Kristin E Mull, Brianna M Sullivan, Ravi Mehrotra

**Affiliations:** 1 Cancer Prevention Program, Division of Public Health Sciences, Fred Hutchinson Cancer Center, 1100 Fairview Avenue North, Seattle, WA, 98109, United States, 1 206 667 5074, 1 206 667 5977; 2 Department of Psychology, University of Washington, Seattle, Washington, United States; 3 Centre for Health, Innovation and Policy Foundation, Noida, Uttar Pradesh, India

**Keywords:** advertisement, dissemination, iCanQuit, India, smartphone applications, smoking cessation, social media, values-based messages

## Abstract

**Background:**

Cigarette smoking is prevalent in many countries worldwide, especially in low- and middle-income countries (LMICs), presenting an urgent public health challenge. Disseminating freely available smoking cessation treatments that effectively decrease cigarette smoking globally is urgently needed.

**Objective:**

Identify the highest impact and most cost-effective values-based social media advertisements to disseminate our smoking cessation smartphone app, “iCanQuit”, among adults living in 7 major cities of India. Values represented in the advertisements included family, relationships, self-care, health, and self-control. Using a quasi-experimental design, we aimed to determine (1) which values-based advertisements had the highest smoking cessation app dissemination reach, as measured by click-through rate (CTR), app installs, and app usage metrics; and (2) which values-based message advertisements were more cost-effective as measured by cost-per-impression, cost-per-click, and cost-per-install. The study population included a selected media market of individuals living in 7 metro cities of India – Delhi, Mumbai, Kolkata, Chennai, Bengaluru, Hyderabad, and Pune – who were exposed to one of 6 social media advertisements from January 16 to May 5, 2024.

**Methods:**

The advertisement campaign design for each of the identified values, based on previous smoking cessation trial data, followed a collaborative iterative process. Advertisements ran sequentially for 16 weeks. Advertisement exposure and app usage data were objectively collected via Google’s Display & Video 360 advertisements campaign management and Firebase app development platforms. Advertisement exposure impact on app engagement was measured via several metrics, including click-through rate (CTR, ie, the likelihood of user clicks on an advertisement after seeing it), the number of app installs (ie, a user opening the app for the first time after downloading it), and the number of app sessions (ie, app usage). Cost efficiency was measured via cost per click and cost per install for each ad.

**Results:**

Overall, the CTR was 5%. The app was installed 5111 times. The average cost per click and cost per app install across all advertisements were US $ 0.006 and US $ 6.43, respectively. The advertisements with the lowest cost per install (range: US $4.83-US $5.16) and highest CTR (between 6% and 9%) focused on the values of family, health, and self-control. Advertisements focused on the values of relationships and self-care had modestly higher levels of engagement.

**Conclusions:**

Advertisements focusing on the values of family, health, and self-control had the highest potential reach at the lowest cost. Overall, these findings provide insights into the reach and cost-effectiveness of values-based messages in social media advertisements, guiding future outreach efforts for population-level dissemination of smoking cessation apps.

## Introduction

Globally, cigarette smoking is a significant public health challenge across many demographic groups [1]. Worldwide, one in 4 adults currently smokes cigarettes and men have almost 5 times higher smoking rates than women [2,3]. India, China, and Indonesia, which are low- and middle-income countries (LMICs), account for 51.4% of the world’s men who smoke. More than half of all smoking-attributed global deaths occurred in China, India, Russia, and the United States combined, with men alone accounting for 75% of these deaths [4].

Quitting smoking is the single most effective method for preventing cigarette smoking-related morbidity and mortality—especially in LMICs [5,6]. Unfortunately, barriers to access to health care, lack of health professionals trained in evidence-based smoking cessation treatments, lack of access to and subsequent usage of such treatments, and transportation-related barriers contribute to smoking cessation programs being out of reach for those who are most in need. Implementing free, remotely accessible digital interventions may improve access to evidence-based smoking cessation programs at the population level.

Digital interventions, such as smartphone apps, offer a newer approach to in-person interventions, with the potential for broader reach and greater acceptance [7,8]. They are freely available on app stores, accessible anytime, and do not always require internet access [9-12]. An efficacious smoking cessation application could be a feasible method for community, clinical, and public health programs to offer scalable smoking cessation services—thereby addressing health inequities in tobacco control.

Our group developed iCanQuit, an Acceptance and Commitment Therapy (ACT)-based smartphone smoking cessation app [13]. Unlike standard interventions for smoking cessation [14], ACT-based smoking cessation interventions teach people to observe, acknowledge, and accept their cravings to smoke rather than avoid them, and focus on life values, instead of expectations, as motivation to quit [13,15]. iCanQuit targets 2 core theoretical processes of ACT, acceptance and values. The acceptance component teaches skills to accept sensations, emotions, and thoughts that cue smoking via distancing from thoughts about smoking, mindfulness, and flexible perspective taking. The ACT-based emphasis on values teaches skills for determining the importance of valued life domains (eg, family, health, and self-care) that motivate quitting smoking.

We tested the efficacy of iCanQuit against a US Clinical Practice Guidelines-based app (QuitGuide) for cigarette smoking cessation in a full-scale randomized clinical trial (RCT) among 2415 adults residing in the United States [16]. iCanQuit was 1.5 times more efficacious than QuitGuide for smoking cessation at 12 months (*P*<.001) [16]. In addition, the intervention’s impact on smoking cessation was mediated by an increase in acceptance of cues to smoke and higher engagement with the app [17]. Although iCanQuit is the only known smoking cessation app proven efficacious for smoking cessation in a full-scale randomized trial, we are aware of no published studies on methods to disseminate any smoking cessation app.

Historically, exposure to antismoking mass media (ie, television) advertisement campaigns has been deployed to disseminate smoking cessation programs at the population level [18-21]. The most used approach is “negative advertising” or the use of advertisements with graphic content that provoke a strong negative emotional response, such as fear, disgust, and sadness about the serious harms of smoking [22]. Tobacco control advertisement campaigns are also more effective when they provide actionable resources for cessation (ie, telephone number for a support line) [23].

An alternative approach to negatively framed advertisements is to design an advertisement campaign with personal values known to motivate behavior change [24]. According to the ACT approach underlying the iCanQuit app, values are “chosen qualities of an action with purpose” [25,26]. Personal values motivate people’s choices, depending on the importance of the value-behavior domain [24]. Personal values can influence behavior change and an individual’s response to health-related information [27,28]. However, little is known about the use of values-based messages in tobacco control population-level advertisement campaigns. A recent study aimed at developing antismoking communication campaigns examined the associations between personal values and tobacco control policies [29]. The most frequently selected values by participants included (1) “assuring my family is safe and secure,” which reflects the value of family and (2) “keeping myself in good health,” which reflects the value of health. These results suggest that values-based messaging is a promising approach in public health communication efforts to increase the adoption of smoking cessation programs at the population level.

Building on this research, we tested the use of values-based messages to disseminate the iCanQuit smoking cessation app at the population level. For this, we selected adults in India, the largest and fastest growing population globally (surpassing China in 2023) [30], with disproportionately high rates of cigarette smoking, especially among men (17.4%, equivalent to >100 million men in India), who have over 6 times higher rates of cigarette smoking than women (2.8%) [4]. Therefore, there is an urgent need to access evidence-based smoking cessation programs.

Given that cigarette smoking is a significant public health challenge in India [31], and to address the need for freely available smoking cessation resources, we tested the dissemination potential of the iCanQuit app to individuals in India. We first conducted user-centered qualitative research to inform the tailoring of iCanQuit to users in India. We then conducted user-centered qualitative research to inform the selection of values-based messages in social media advertisement campaigns. Subsequently, we conducted a pilot social media advertisement campaign to determine the most efficient and cost-effective strategies for reaching a broad audience of adults who smoke. The 16-week advertisement campaign aimed to compare the reach of 5 personal values-based message advertisements (ie, family, relationships, self-care, health, and self-control) known to motivate behavior change in promoting installation and usage of the iCanQuit app [24,32].

The study’s main objective was to identify the highest impact values-based social media advertisements to disseminate our smoking cessation smartphone app, “iCanQuit,” among adults living in 7 major cities of India. Using a quasi-experimental design, the goals were to (1) determine which values-based advertisements had the highest smoking cessation app dissemination reach, as measured by click-through rate (CTR), app installs, and app usage metrics; and (2) determine which values-based message advertisements were more cost-effective as measured by cost-per-impression, cost-per-click, and cost-per-install.

## Methods

### Overview

The main objective of this quasi-experimental comparative study was to identify the highest reaching and most cost-effective values-based social media advertisement to disseminate a smoking cessation app already proven to be efficacious to help adults quit cigarette smoking [16]. The visual advertisement messaging was focused on various personal values hypothesized to motivate adults to quit smoking. In the context of this campaign, we tested various personal values messaging that may motivate viewers exposed to the advertisements to install the app, thereby promoting app engagement.

Adults in a selected media market in India were exposed to one of 6 advertisements that ran sequentially from January 16, 2024, to May 5, 2024 (Table 1). The user sees the ad, finds the message interesting, clicks on it, and is directed to the advertisement’s website (ie, the app store iCanQuit page). Over the course of 16 weeks, the digital advertisement campaign sequentially deployed each advertisement. The generic control advertisement ran for about 6 weeks, while each values-based advertisement ran for about 2 weeks.

**Table 1.** Advertisement campaign active dates.

Type of value-based advertisement	Dates advertisement was active	Number of days
Generic control ad	01/16/2024-02/26/2024	42
Family	02/22/2024-03/12/2024	20
Relationships	02/22/2024-02/26/2024 03/16/2024-03/23/2024	13
Self-care	03/25/2024-04/07/2024	14
Health	04/08/2024-04/22/2024	15
Self-control	04/23/2024-05/05/2024	13

### Priority Population

The study population included individuals living in 7 metro cities of India – Delhi, Mumbai, Kolkata, Chennai, Bengaluru, Hyderabad, and Pune—as the test areas due to their high population density as well as cultural and linguistic diversity [33].

### Values-Based Visual Advertisements

Personal values were identified based on empirical research data from previous digital smoking cessation intervention trials conducted by our group [16,34]. We selected the most frequently reported personal values that participants cited as motivations for quitting cigarette smoking. These values include: (1) family, (2) health, (3) money, (4) spirituality, (5) relationships, (6) pets, (7) self-growth, and (8) friends.

The advertisement campaign design for each of the identified values, based on previous smoking cessation trial data, followed a collaborative 2-step process. Step 1 involved an expert-guided development of values-based messages to motivate adults who smoke to quit smoking. Our research and clinical team of investigators and colleagues with expertise in ACT worked together to generate values-based messages based on their expertise in delivering behavior-based interventions [13,26]. Step 2 involved developing visual advertisements for each of the 5 selected messages through an iterative design process. We collaborated with a vendor specializing in visual advertisement creation (GroupM) [35], ensuring each advertisement was aligned with a specific message.

**Figure 1.**
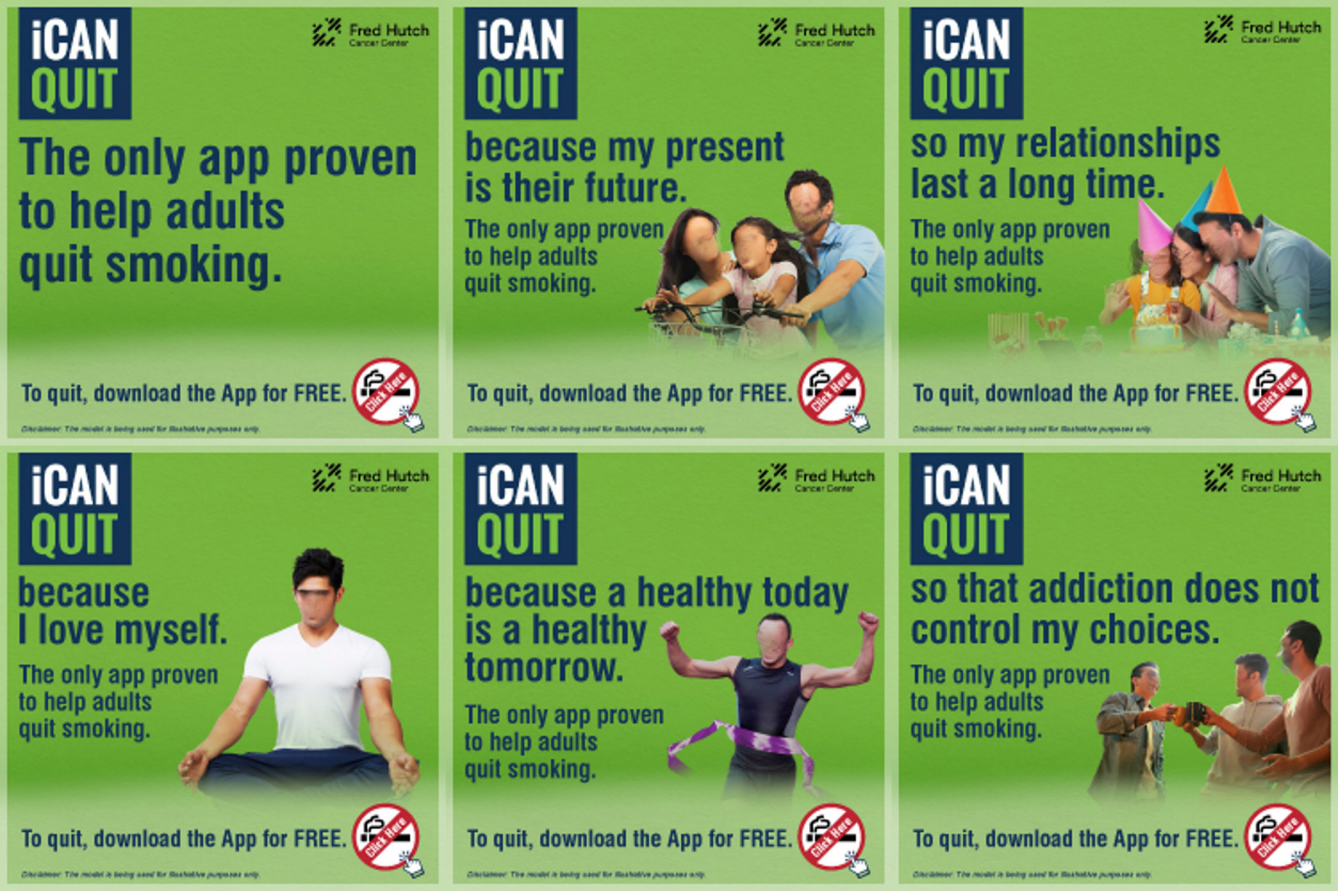
Visual advertisements used in the campaign. Top row, from left to right: generic, family, relationships. Bottom row, from left to right: self-care, health, self-control.

Visual advertisements were created to align with the 5 personal values of family, relationships, self-care, health, and self-control. A sixth generic control advertisement that did not correspond to a specific value served as a reference (Figure 1). Advertisements ran in the English language. Each visual advertisement ran in 4 mobile-friendly image sizes: 250 × 250, 300 × 250, 320 × 480, and 320 × 50 pixels.

### Conceptual Model

**Figure 2.**
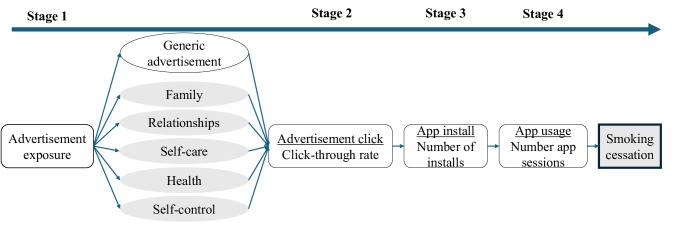
Conceptual model: values-based messages in social media advertisement.

We hypothesized that exposure to values-based messages via social media advertisements results in app engagement via a 4-stage process (Figure 2). In stage 1, adults in a selected media market in India are exposed to one of 6 advertisements—a generic control advertisement and 5 values-based advertisements—that run sequentially. In stage 2, the values-based messaging in the ads, which is theorized to resonate with adults who smoke, leads to advertisement clicks, which guide users to download the app. In stage 3, users download and install the app on their smartphones. In stage 4, users engage with the app, and it is theorized that engagement with the smoking cessation app leads to smoking cessation given that engagement is a strong predictor of smoking cessation [17,36]. Note that smoking cessation is not an outcome of this study and thus was not measured.

### Data Collection Overview

Data were objectively collected via Google’s Display & Video 360 (DV360) advertisements campaign management and Firebase app development platforms. Advertisement exposure and advertisement clicks were measured through DV360, while app installs and app usage metrics (user usage of the iCanQuit app once installed) were tracked via Firebase. Once an app was successfully installed, the user was linked to the advertisement they clicked as captured via the DV360 platform.

#### Stage 1. Advertisement Exposure

We used Google’s Display & Video 360 (DV360) platform for content-oriented advertising. DV360 is an advertising platform within the Google Marketing Platform, which is designed for managing display and video advertisement campaigns. DV360 seamlessly integrates advertisements into the user’s web browsing experience through tailoring and real-time bidding processes, ensuring relevant advertisements are shown to the right users at the right time. The details of each step in this process are outlined in Table 2.

**Table 2.** Steps in visual advertisement exposure through DV360.

Step	Description
Browsing online	A user browses the web by visiting a blog or web page.
Real-time bidding and advertisement request sent	As the page loads, it sends an advertisement request to an advertisement exchange (ie, DV360 platform).
User profile matching and DV360 bids	DV360 identifies the user as “interested” or “high intent” based on the set of content-oriented parameters and places a bid. Advertisements are also triggered on web pages that include relevant keywords (ie, content-oriented keywords).
Auction and advertisement selection	DV360 wins the auction, and an advertisement is selected.
Advertisement display	The advertisement appears in a banner slot on the blog or web page that the user is browsing.
Post advertisement user interaction	The user sees the ad, finds the message interesting, clicks on it, and is directed to the ad’s website (ie, the app store iCanQuit page).

Advertisements were tailored to affinity and in-market audience segments, as well as to users of 48 competitor smoking cessation apps, for their likely interest in smoking cessation to maximize the impact and cost-efficiency of the campaign. Affinity groups used for advertisement tailoring included the following: food and dining, frequently dines out, frequently eats breakfast out, frequently eats lunch out, frequently eats dinner out, coffee shop regulars, fashionistas, beauty mavens, shutterbugs, technophiles, frequently attends live events, pop music fans, theater aficionados, and media and entertainment. In-market segments selected included makeup and cosmetics, soccer tickets, party supplies and planning, event planning services, business financial services, and vacation packages. Advertisements were compared descriptively on several metrics. For instance, they were compared on impressions, which is the number of times a visual advertisement is displayed to a user on a webpage or blog.

The full advertisement campaign ran from January 16, 2024, to May 5, 2024 (Table 1). Over the course of 16 weeks, the digital advertisement campaign sequentially deployed each ad. The generic control advertisement ran for about 6 weeks, while each values-based advertisement ran for about 2 weeks.

#### Stage 2. Advertisement Clicks

Clicks are the number of times users interact with an advertisement by clicking on it. CTR is the ratio of clicks to impressions, expressed as a percentage. It measures the likelihood of users clicking on an advertisement after being exposed to it, indicating interest in the message.

#### Stage 3. App Installs

App installs are the number of times an app is successfully downloaded and installed on a device. The conversion rate is the ratio of installations to clicks. The cost per install is the average cost for each app installation, calculated by dividing the total advertisement campaign cost by the number of app installs. To monitor app installs and assess the impact of the advertisement campaign on app usage metrics as a measure of app engagement, the mobile measurement platform, Firebase, was integrated with the advertisement serving platform Campaign Manager DV360. Firebase is a Backend-as-a-Service (BaaS) platform developed by Google that provides a suite of tools and services to help developers build, deploy, and manage applications, particularly mobile and web apps, with features like real-time database, authentication, and cloud storage. This integration facilitated the tracking of app installations and app usage metrics described below.

#### Stage 4. App Usage Metrics

Once the iCanQuit app was successfully installed, all in-app activity was tracked by Firebase. Several usage metrics were summarized for the first 30 days for each app user, including the (1) number of app sessions, (2) time per session, (3) unique days of use, and (4) length of use from the first to last session. Smoking status and cessation were not measured.

### Statistical Analysis

Users were linked to the advertisement they viewed through advertisement tracking within Firebase, the Google platform used to track app installs and app usage. When an app was successfully installed, the user was assigned an advertisement tracking ID corresponding to the Google advertisement clicked to initiate the app download. For users who had advertisement tracking disabled, we assigned the advertisement campaign based on the installation date, selecting the campaign closest to that date.

Given the quasi-experimental design, there were no inclusion or exclusion criteria nor missing data. However, there were 34 users who had advertisement tracking features disabled and installed the app between February 22‐26, 2024, when 3 advertisements were running simultaneously. As it was impossible to assign these users to a specific advertisement, they were excluded from the analysis.

To compare the values-based advertisements on advertisement metrics, we used chi-square tests for CTR and cost per install and ANOVA for the number of installs per day. Advertisements were compared on app usage counts using negative binomial models for the number of app sessions, unique days of use, and length of use, given their heavily right-skewed distributions. Finally, advertisements were compared on time per session using ANOVA.

### Ethical Considerations

All study procedures, protocols, and procedures for the advertisement campaign were approved by the Fred Hutchinson Cancer Center’s Institutional Review Board and determined to be a nonhuman subject research since we did not interact directly with individuals, nor collect any personally identifiable information.

## Results

### Advertisement Clicks

The advertisement campaigns resulted in a total of 119,258,689 impressions and 5,635,114 clicks (Table 3). CTR significantly varied across advertisements (*P*<.001), averaging 5% across ads. The relationships and health values-based advertisements had higher CTRs, at 9% each.

**Table 3.** Advertisement metrics.

Variables	Type of ad							*P* value
	Overall	Generic	Family	Relationships	Self-care	Health	Self-control	
Number of impressions	119,258,689	25,939,433	21,783,538	14,292,366	26,077,665	14,093,203	17,072,484	NA^a^
Number of clicks	5,635,114	911,843	1,261,810	494,136	153,747	1,285,553	1,528,025	NA
Click-through rate^b^, %	5%	4%	6%	3%	1%	9%	9%	<.001
Number of app installs	5111	1149	1192	509	346	946	969	NA
Average number of installs per day, mean (SD)	46.0 (28.3)	27.4 (13.8)	59.6 (27.6)	39.2 (19.2)	24.7 (19.9)	63.1 (30.5)	74.5 (17.2)	<.001
Conversion rate^c^, %	0.09%	0.13%	0.09%	0.10%	0.23%	0.07%	0.06%	<.001

a NA: not applicable

b Click-through rate = Number of clicks/Number of impressions.

c Conversion rate = Number of app installs/Number of clicks.

### App Installs

The advertisement campaigns resulted in a total of 5111 app installations (app installs; Table 3). App installs per day varied significantly across advertisements (*P*<.001). The self-control and health value-based advertisements averaged the greatest number of installs per day, with means of 74.5 (SD=17.2) and 63.1 (30.5), respectively. The conversion rate, or the ratio of installations to clicks, also varied significantly between advertisements (*P*<.001). The self-care value-based advertisement had the highest conversion rate of 0.23%, while the self-control value-based advertisement had the lowest conversion rate of 0.06%.

### App Usage Metrics

The number of app sessions, minutes spent per session, unique days of use, and days from the first to last session were summarized for the first 30 days for each of the 5111 app users in India. Summary usage statistics are given in Table 4 for all app users combined, and separately by advertisement viewed. Most app usage metrics were similar between values-based advertisement campaigns, except number of app sessions varied significantly across campaigns (*P*=.04). The relationships value-based advertisement averaged a higher number of app sessions per user (mean=2.3, median=1). The mean length of app use in the first 30 days was 9.3 (SD=15.8) days

**Table 4.** App usage metrics.

Usage metric, mean (SD)	Overall (n=5111)	Type of advertisement						*P* value
		Generic n=1149	Family n=1192	Relationships n=509	Self-care n=346	Health n=946	Self-control n=969	
Number of app sessions	2.1 (2.7)^a^	2.2 (3.1)^a^	2.0 (2.2)^a^	2.3 (3.3)^a^	2.2 (3.9)^a^	2.1 (2.3)^a^	2.1 (2.4)^a^	.04
Time per session, minutes	0.9 (2.0)^b^	0.9 (1.9)^b^	0.9 (1.9)^b^	1.0 (1.7)^b^	0.8 (1.8)^c^	0.8 (1.8)^b^	0.9 (2.6)^b^	.85
Unique days of use, days	1.8 (1.9)^a^	1.9 (2.1)^a^	1.8 (1.6)^a^	2.0 (2.1)^a^	1.9 (2.5)^a^	1.8 (1.7)^a^	1.8 (1.7)^a^	.17
Length of use from first to last session, days	9.3 (15.8)^a^	9.3 (16.0)^a^	9.4 (15.9)^a^	10.2 (16.6)^a^	10.3 (16.7)^a^	9.0 (15.4)^a^	8.5 (15.2)^a^	.14

a Median=1.

b Median=0.3.

c Median=0.2

d During the first 30 days after downloading the app.

### Advertisement Budget and Cost

The campaign budget was evenly divided across the 5 values-based advertisements to ensure equal exposure and allow for unbiased results. The total budget for the iCanQuit India initiative was Indian Rupees 26 lakhs, equivalent to 2,600,000 rupees or US$ 34,211 (exchange rate on February 27, 2024). The total campaign media advertisement spend was US $32,847.18. The 3 top-performing advertisements in terms of cost per install and number of installations were family, health, and self-control value-based ads, while the worst-performing advertisements were relationships and self-care value-based advertisements. The full cost breakdown by advertisement is given in Table 5. The average cost per install across all advertisements was US $6.43; the average cost per install across the family, health, and self-control value-based advertisements was US $4.97, with the family value-based advertisement having the lowest cost per install at US $4.83.

**Table 5.** Advertisement budget and cost.

Variables	Type of advertisement						
	Overall	Generic	Family	Relationships	Self-care	Health	Self-control
Advertisement spending, USD	$32,847.18	$6016.61	$5756.81	$4976.20	$6400.43	$4885.89	$4811.24
Cost-per 1000 impressions, USD	$0.275	$0.232	$0.264	$0.348	$0.245	$0.347	$0.282
Cost-per-click, USD	$0.006	$0.007	$0.005	$0.010	$0.042	$0.004	$0.003
Cost-per-install, USD	$6.43	$5.24	$4.83	$9.78	$18.50	$5.16	$4.97

## Discussion

### Principal Findings

The success of the iCanQuit values-based advertisement campaign was measured by its ability to effectively communicate personal values hypothesized to motivate change in behavior (ie, smoking cessation) and its yield of app installs by the selected audience (users in India). The advertisement campaign generated approximately 119 million impressions and 5.6 million clicks with an average click-through rate of 5% total. This click-through rate was greater than industry standards, which range between 0.5% and 1% [37]. The campaign resulted in approximately 5111 app installs, with reach (click-through rate and conversion rate) and cost efficiency of the campaign varying across advertisements.

The self-control value-based advertisement achieved the highest reach and cost-effectiveness, followed by the health value-based advertisement, suggesting that self-control and health are key motivators for smoking cessation among people in India. In contrast, the self-care and relationships value-based advertisements showed relatively low reach and cost-effectiveness. The self-care value-based advertisement had the highest conversion rate of all advertisements, but its absolute volume of conversions remained the lowest. We are unsure why the self-care advertisement did not perform well, though we believe the advertisement’s high conversion rate is due to its absolute number of impressions. Specifically, the self-care advertisement had nearly twice as many impressions (26,077,665) as the health advertisement (14,093,203), even though both advertisements ran for a similar number of days (14 and 15 days, respectively). There are a few plausible explanations for this. First, it is possible that the algorithm favored the self-care advertisement more than the health advertisement, giving it more exposure. Second, the self-care advertisement may have won more advertisement auctions, which is an iterative process in which both tailoring and real-time bidding processes ensure relevant advertisements are shown to the right users at the right time. For instance, if the self-care advertisement initially generated a higher click-through rate, the algorithm would predict strong performance and therefore show it more often. In other words, the advertisement received more screen time because it was perceived as more engaging and better suited for the audience.

While the relationship values-based advertisement resulted in the greatest engagement with the app, the advertisement had relatively low reach and cost-effectiveness. Finally, while the generic control advertisement had a relatively low cost per install (US $5.24), this advertisement yielded a total of 1149 installs in 6 weeks compared with the family value-based advertisement which yielded 1192 in half the time.

### Importance of Findings

This is the first study, to our knowledge, to draw a direct comparison of the reach and cost-effectiveness of values-based advertisement campaigns for disseminating a smoking cessation app at the population level. We found that advertisements focusing on the values of self-control and health yielded the highest reach at the lowest cost, suggesting potential for broad dissemination and scalability. The high performance of self-control-based messages indicates that the ability to regulate urges to smoke as a strategy to quitting smoking may resonate with adults in India. The health-based messaging confirms previous research suggesting health is an important motivator to quitting smoking [38].

### Comparison With Previous Work

To put the findings of this study in context, the only known previous study of a mobile-based smoking cessation program is a media campaign for SmokeFreeTXT (SFT), a text messaging program for adolescents in the United States [39]. This was a nationwide campaign that included traditional (television and radio), online, and social media outreach with action-based advertisement messaging. The SFT social media campaign resulted in a click-through rate of 0.14%, as compared with 5% in our study. In terms of cost, the cost-per-click in the SFT social media campaign was US $0.63 compared with a cost-per-click of US $0.0006 in our study. These results suggest that our social media campaign was more effective in reach and cost among adults in India. The SFT measure of engagement was the number of new website sign-ups, which was 2384 new sign-ups in 8 weeks. In comparison, our study achieved 5111 app installs in a period of 16 weeks, demonstrating the feasibility and dissemination potential of our values-based social media campaign to LMICs in a relatively shorter period of time.

Our work extends previous research by demonstrating the feasibility of a social media advertisement campaign as a cost-effective strategy to disseminate a smoking cessation app in an LMIC such as India. While most tobacco control advertisement campaigns, whether digital or traditional, have been conducted and evaluated in high-income countries (ie, the United States of America, Canada, Australia, and the United Kingdom) [40], our study focused on a priority population in an LMIC. Second, antitobacco campaigns have historically focused on the negative health consequences of smoking, whereas our study provides first-time data on the potential reach and cost-effectiveness of a mobile-based visual advertisement campaign for a smoking cessation app that focused on personal values known to motivate behavior change. Finally, beyond providing data on advertisement exposure, our study also extends this literature by further evaluating app usage metrics provided, an overall measure of engagement, known to contribute to smoking cessation [17,36].

The results also offer specific insights for future media advertisement campaigns. First, close monitoring of advertisement campaign performance is essential. While some advertisements may initially show higher engagement and be favored by platform algorithms, the click-through rates need to be put in perspective with other important metrics. For instance, these early signals, such as the click-through rate, must be interpreted alongside other outcomes such as the conversion rate and cost-effectiveness. Second, greater exposure does not necessarily translate to better performance. For example, although the generic advertisement ran longer (6 weeks vs 2 weeks on average) and had a lower cost per impression, it yielded modest click-through rate and conversion rate compared to shorter values-based advertisement campaigns. Finally, the resonance of advertisement content with the target audience matters. Tailoring advertisement messages to the cultural and motivational context of the audience is critical, and user-centered research in the intended market is a key step for campaign success.

### Limitations

This study has several key limitations. First, the nonrandomized design introduces the potential for selection bias, as users who engaged with a particular type of advertisement may differ systematically from those exposed to other advertisements in this study. In addition, user characteristics were not collected, meaning unmeasured confounders, such as socioeconomic status or smoking behaviors, could have influenced both advertisement engagement and app usage. Second, individuals may have seen multiple advertisements or viewed the same advertisement multiple times, and thus, previous exposure to earlier advertisements and their potential influence on engagement could not be measured. Third, in rare cases when multiple advertisement campaigns ran simultaneously, it was not possible to objectively determine which advertisement(s) an individual was exposed to if they had advertisement tracking turned off (n=34 users excluded, 0.7% of the sample). Fourth, findings are limited to individuals in 7 selected urban areas in India comprising a total population of 107.3 million people. Replication is needed in other regions, including rural areas in India and other countries. Finally, this study did not evaluate the impact of advertisements on key smoking cessation outcomes, such as intentions to quit, quit attempts, or sustained cessation. Future research should assess whether these types of advertisement campaigns ultimately lead to smoking cessation.

### Conclusions

The results of this study indicate that exposure to advertisements focusing on the values of family, health, and self-control had the highest potential reach at the lowest cost. Overall, these findings provide insights into the reach and cost-effectiveness of values-based messages in social media advertisement, guiding future outreach efforts for population-level dissemination of smoking cessation apps.

## Abbreviations

ACT: acceptance and commitment therapy
CTR: click-through rate
LMICs: low- and middle-income countries
RCT: randomized controlled trial
SFT: SmokeFreeTXT
